# coPERCIST: AI-assisted PET-CT response assessment

**DOI:** 10.1007/s00259-025-07614-3

**Published:** 2025-10-22

**Authors:** Elin Trägårdh, Måns Larsson, Olof Enqvist, Tony Gillberg, Malene Grubbe Hildebrandt, Lars Edenbrandt

**Affiliations:** 1 Department of Clinical Physiology and Nuclear Medicine, Skåne University Hospital, Inga Marie Nilssons G 49, 205 02 Malmö, Sweden; 2 Department of Translational Medicine and Wallenberg Center for Molecular Medicine, Lund University, Malmö, Sweden; 3 Eigenvision AB, Lund, Sweden; 4 Chalmers University of Technology, Gothenburg, Sweden; 5 Slicevault AB, Malmö, Sweden; 6 Department of Clinical Research, University of Southern Denmark, Odense, Denmark; 7 Department of Nuclear Medicine, Odense University Hospital, Odense, Denmark; 8 Department of Molecular and Clinical Medicine, Gothenburg University, Gothenburg, Sweden

**Keywords:** PERCIST 1.0, Treatment response, Cancer, PET-CT

## Abstract

**Purpose:**

The PET Response Criteria in Solid Tumours (PERCIST) 1.0 provides a standardized framework for evaluating treatment response using [^18^F]fluorodeoxyglucose ([^18^F]FDG) positron emission tomography – computed tomography (PET-CT), but its clinical use is hindered by manual complexity. This study presents coPERCIST, an artificial intelligence (AI)-assisted module integrated into the RECOMIA platform that semi-automates and streamlines PERCIST analysis.

**Methods:**

coPERCIST performs organ segmentation and automates key steps of the PERCIST workflow, including background activity quantification, lesion detection, SULpeak calculation, and longitudinal lesion comparison. A novel image alignment method using organ-specific transformations and uncertainty estimation enables accurate lesion tracking over time. The system was evaluated in 58 oncological patients, each with two PET-CT scans. Up to three measurable lesions per patient were analysed.

**Results:**

The AI-suggested liver and aorta volume of interest for threshold calculation were correct in all baseline and follow-up studies. Follow-up studies were classified as progressive metabolic disease (PMD) in 38 cases, stable metabolic disease (SMD) in 16, and partial metabolic response (PMR) in 4. Of 130 lesions evaluated, anatomical alignment was accurate in all cases, and pairwise SULpeak quantification was accurate in 95%. Pairwise SULpeak quantification failed in seven lesion pairs due to proximity to other lesions or misclassified physiological uptake. Review time was less than one minute for most cases.

**Conclusion:**

This study demonstrates the feasibility of AI-assisted PERCIST evaluation for [^18^F]FDG PET-CT, showing promising accuracy. coPERCIST offers potential for reproducible response assessment and supports future multicentre validation. It is freely available to researchers via the RECOMIA platform.

**Supplementary Information:**

The online version contains supplementary material available at 10.1007/s00259-025-07614-3.

## Introduction

Positron emission tomography combined with computed tomography (PET-CT) offers a powerful tool for treatment response assessment in oncology, but interpretation often lacks the objectivity and reproducibility necessary for consistent clinical decisions and reliable research endpoints. The PET Response Criteria in Solid tumours (PERCIST) 1.0 framework addresses this by providing a standardized, quantitative approach for evaluating metabolic response in [^18^F]fluorodeoxyglucose ([^18^F]FDG)-PET [1]. Despite its advantages, applying PERCIST remains time-consuming and labour-intensive, requiring careful lesion identification, segmentation, and measurement across time points. This complexity limits its use in routine care and large-scale studies. Advances in artificial intelligence (AI) offer the opportunity to automate key components of the PERCIST workflow, improving efficiency while maintaining, and potentially enhancing, objectivity and reproducibility.

Most PET-CT workstations are designed for routine visual analysis. While many platforms offer advanced visualization and quantification capabilities, few provide dedicated tools or streamlined workflows for rigorous, quantitative analyses like PERCIST. Applying PERCIST requires complex steps: defining volumes of interest (VOI) in the liver, aorta, and target lesions of varying size and shape; computing background uptake; performing lean body mass–corrected standardized uptake value (SUL) calculations; applying thresholds; and excluding normal physiological tracer activity in regions such as the brain, heart, and urinary system [1]. These tasks, when performed manually, are laborious and prone to variability, posing a barrier to the wider adoption of PERCIST in clinical care and research. AI can overcome these barriers by enabling automated, reproducible processing of PET-CT data. For longitudinal response assessment, robust alignment of serial PET-CT scans is essential. Various alignment approaches have been explored, including image registration, graph-based methods, landmark detection, and deep neural networks [2–13]. While AI is increasingly used in nuclear medicine research for tasks like image reconstruction, segmentation, quantification, and reporting [14–23], most existing work focus on single time-point analysis rather than longitudinal comparisons.

Some studies have addressed longitudinal whole-body PET-CT alignment for tumour tracking or therapy response assessment [2–4, 6, 7, 13]. Jemaa et al. [24] recently developed an automated Lugano response assessment pipeline for non-Hodgkin lymphoma using deep learning-based segmentation, image registration, and rule-based response classification. However, no comparable AI-assisted platform exists for PERCIST evaluation despite its relevance across a broad range of solid tumours.

This work aimed to develop coPERCIST, an AI-assisted module for streamlined, semi-automated implementation of PERCIST in serial [^18^F]FDG PET-CT studies.

## Method

### Overview of coPERCIST

The method was designed to automate key steps of the PERCIST workflow, including alignment of serial scans, quantification of background activity, lesion detection, SULpeak calculation and final response assessment in a consistent and reproducible manner. Manual interactions included accepting the VOI location for background activity (and threshold value), verifying lesion selection, and verifying new or progressive lesions.

coPERCIST was implemented as an extension of the existing RECOMIA platform, a cloud-based research environment for medical image analysis. Available to academic researchers since 2020, RECOMIA provides secure infrastructure for image de-identification, transfer, visualization, and analysis [21]. It includes deep learning-based tools for organ segmentation and quantification, which support the PERCIST analysis pipeline described below.

**Fig. 1:**
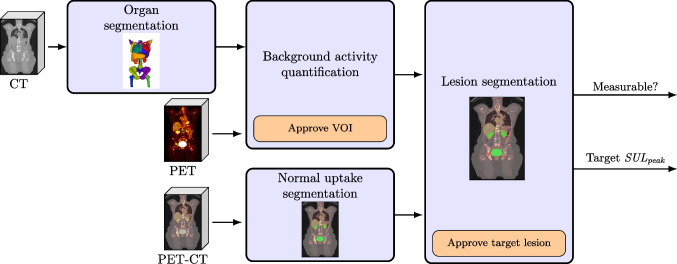
Schematic overview of coPERCIST for processing the baseline study. Light blue boxes indicate fully automatic steps, while orange boxes indicate steps that require manual input

**Fig. 2:**
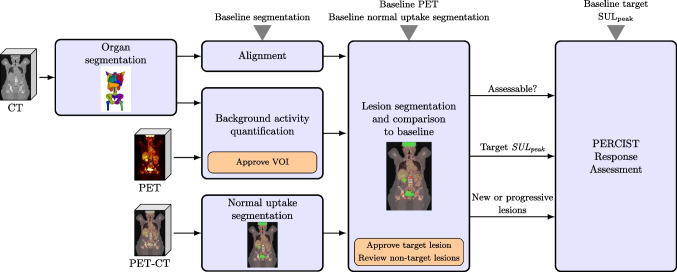
Schematic overview of coPERCIST for processing the follow-up study. Light blue boxes indicate fully automatic steps, while orange boxes indicate steps that require manual input

The main steps of the approach were as follows (visualized in Figs. 1 and 2):

#### Baseline analysis

- **Segmentation of organs and normal PET activity:** AI-based fully automatic segmentations of organs and normal PET activity were used in the following steps: quantification of background activity, lesion classification, and alignment of serial PET-CT studies.
- **Background activity:** Liver and aorta background activity was calculated using automatic organ segmentations, per PERCIST [1]. Measurement of background activity was used to establish the threshold for lesions and to verify that two PET-CT studies were comparable. According to PERCIST, the liver is normally used as background activity, but if a patient has metastatic disease such that the VOI could not be used without including tumour, the aortic VOI is used instead.
- **Lesion segmentation:**
  - Based on the automatically calculated threshold, all volumes (“hotspots”) with higher SULpeak in the PET image were detected.
  - The hotspots were classified as either lesions, marked in red, or normal activity, marked in green, based on an AI-segmentation of normal activity. In each volume, a spherical VOI of 1 cm3 was positioned at the highest SULpeak.
  - Each hotspot was ordered with respect to the SULpeak in a list.
- **Hotspot inspection:** The user reviewed the automatically labelled hotspots to confirm or correct the classification. The highest confirmed malignant SULpeak was recorded and used as the reference for treatment response evaluation.

#### Follow-up analysis

**Fig. 3:**
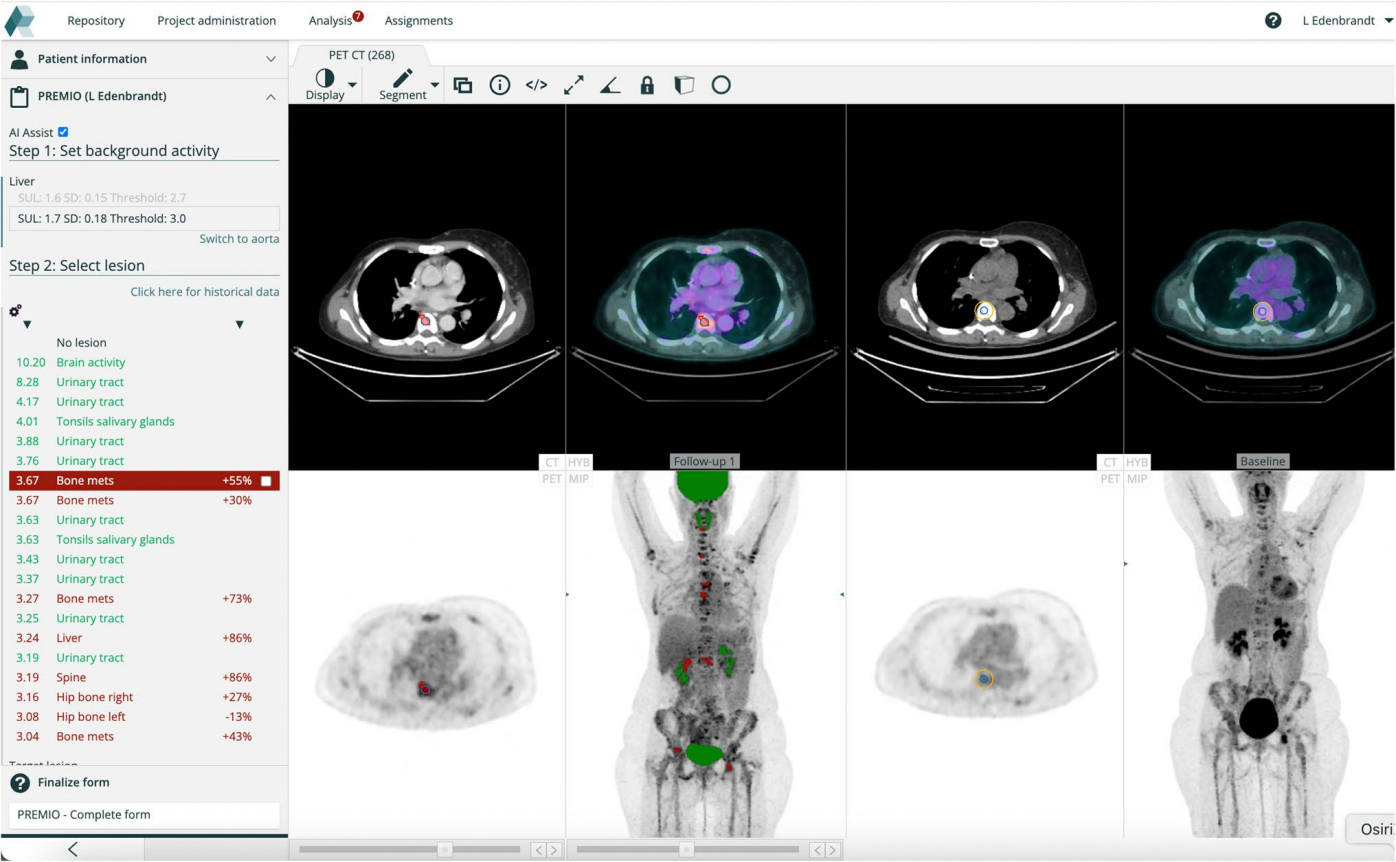
The coPERCIST module on the RECOMIA platform with baseline (right) and follow-up PET-CT (mid). All hotspots are compared between the scans and sorted according to SULpeak or percentage change in the left panel. Normal uptake is shown in green and lesions in red

For follow-up studies, in addition to the steps described above, the following steps were also carried out:

- **Alignment to baseline scan.** Using a method that will be described in detail below, a transformation field was computed that assigned each pixel in the follow-up scan to its corresponding pixel in the baseline scan.
- **Lesion segmentation:** As described above with the addition of that for each lesion, the relative change in SULpeak compared to the baseline scan was computed. The increases or decreases in activity were displayed as percentage changes and the corresponding region in the baseline scan was automatically highlighted. The user reviewed the automatically labelled hotspots to confirm or correct the classification with the aid of the percentage change, determined whether the lesion represented a new lesion, a lesion with unequivocal progression, or neither. Figure 3 illustrates how this step is performed in the module.
- **Automatic PERCIST response assessment:**
  - If no lesions were indicated in the follow-up scan, a complete metabolic response (CMR) was registered.
  - If no new lesions were indicated, no lesion had increased unequivocally, and the target SULpeak had decreased by at least 30% (and at least 0.8 SUL units), partial metabolic response (PMR) was registered.
  - If the target SULpeak had increased more than 30% (and at least 0.8 SUL units) or any individual lesion had increased unequivocally, or new lesions were indicated, progressive metabolic disease (PMD) was registered.
  - If none of the above held then stable metabolic disease (SMD) was registered.

### Organ segmentation

The baseline and follow-up CT images were automatically segmented using two deep learning models. Most organs were segmented with an updated version of Organ Finder [25], capable of identifying 56 anatomical structures.

In addition to the organs segmented by Organ Finder, individual vertebrae were segmented using a custom two-stage pipeline inspired by Spine-transformers [26]. First, a CNN detects vertebral centre points; then a 3D U-Net generates instance-level vertebra segmentations centred at these locations. Both vertebrae models were trained on a combined dataset of 856 CT volumes, including 100 cases from the VerSe dataset [27] and 756 PET-CT scans from cancer patients, the latter annotated using TotalSegmentator [28] and manually reviewed. Only vertebrae fully visible in the field of view were included. More details can be found in the Supplementary material.

### Normal activity segmentation

An AI tool was trained to segment normal activity in PET studies. The training was based on 873 PET-CT studies from the FDG-PET-CT-Lesions dataset [29] with corresponding manual segmentation of activity related to the intestinal and urinary tracts as well as uptake in the brain, heart, extraocular muscles, salivary glands, tonsils, vocal cords, and testes. More details can be found in the Supplementary material.

### Background activity

To quantify the background activity, the SULmean, standard deviation (SD), and threshold were calculated for the liver and aorta using organ segmentations by the Organ Finder [25] with a spherical VOI (3-cm diameter) for the liver and a cylindrical VOI (1-cm diameter, 2-cm height) in the descending aorta. The exact placement was determined to avoid focal lesions and noise, see supplemental material for details. Lean body mass was computed as suggested by James et al. [30] which is recommended by PERCIST [1].

### Longitudinal SULpeak comparison

For each lesion identified in the follow-up scan, the relative change in SULpeak compared to baseline was calculated. To enable this comparison, baseline and follow-up images were spatially aligned. Additionally, an alignment uncertainty was estimated for each voxel.

For a lesion segmented in the follow-up image, the voxel location corresponding to the SULpeak was mapped to the baseline image using the transformation derived from image alignment. Within a sphere centred on the mapped voxel, with a radius defined by the estimated uncertainty, the highest non-physiological SULpeak value was extracted. This value was then compared to the SULpeak of the lesion in the follow-up image.

### Alignment method

Our method aligned a source PET image to a target PET image using organ segmentations derived from their corresponding CT images. The method consisted of two main steps:

1. **Organ-wise transformation estimation.** Each organ segmented in both source and target CTs was aligned independently. Rigid organs (e.g., bones) were aligned using rigid transformations, while soft-tissue organs (e.g., liver, lungs) were aligned using affine transformations [31] to account for local deformations.
2. **Interpolation and extrapolation.** Organ-specific transformations were propagated throughout the full image domain to obtain a dense displacement field.

The final output of the alignment method was a dense displacement field that spatially mapped the source PET image to the target PET image.

#### Organ-wise transformation estimation

##### Surface extraction and correspondence

Organ surfaces were extracted from both source and target CT segmentations using the marching cubes algorithm [32]. Correspondences between surfaces were estimated using the iterative closest point (ICP) algorithm [33]. Initialization was performed by aligning the surface point closest to the image centre in each scan, which improved robustness in cases where centre-of-mass initialization failed due to differences in field of view.

During each ICP iteration, the current transformation was applied to the source surface, nearest-neighbour correspondences were established, and the transformation was updated accordingly.

##### Rigid transformation

For rigid organs, correspondences were filtered to remove outliers by discarding pairs whose distance exceeded four times the median. The remaining point pairs were then used to compute the optimal rigid transformation, consisting of a rotation and a translation, using the Kabsch algorithm [34].

##### Affine transformation

For soft-tissue organs, the correspondences were instead used to compute an affine transformation. This transformation allowed for rotation, translation, scaling, and shearing, and was estimated by solving a least-squares problem in homogeneous coordinates.

#### Interpolation and extrapolation

To extend the organ-specific transformations to the entire image volume, Laplace’s equation was solved with the organ transformations as Dirichlet boundary conditions:

$${\Delta }^{2}u\left(x\right) = 0$$

where u(x) is the displacement vector at voxel x. Voxels within segmented organs were fixed to their estimated transformations while all other voxels were initialized to the mean of all organ transformation. To solve the equation, iterative updates were performed using the Jacobi method with a six-voxel neighbourhood until convergence.

To prevent anatomically implausible propagation (e.g., arm transformations affecting abdominal voxels), interpolation was performed independently in six regions: head, torso, left/right arms, and left/right legs. This regional decomposition preserved anatomical boundaries and improved robustness to variable body positioning (e.g., arms positioned alongside vs. across the torso). The alignment method was evaluated on a dataset consisting of 181 patients who underwent PET-CT imaging at 1 and 2 h after injection of [^18^F]PSMA-1007 and is described in detail in the Supplementary material.

#### Alignment uncertainty estimation

Alignment uncertainty was estimated based on organ-level segmentation results. For each image pair, TotalSegmentator was used to segment a predefined set of organs in both the baseline and follow-up scans. The images were then aligned, and the resulting transformation was applied to propagate the baseline organ segmentations to the follow-up image. The Hausdorff distance [35] between the propagated and actual segmentations was calculated for each organ, and organs were grouped according to uncertainty levels based on these distances.

For a voxel located within an organ, the uncertainty was defined as the 75th percentile of the Hausdorff distances for that organ. For voxels outside segmented organs, uncertainty increased linearly with distance from the nearest organ boundary, at a rate of 0.2 mm per mm.

### Evaluation

A test group of 58 patients (27 with melanoma, 19 with breast cancer and 12 with colorectal cancer) was used to evaluate the PERCIST method of the RECOMIA platform. Inclusion criteria were metastatic disease, two [^18^F]FDG PET-CT scans, and measurable disease (defined as at least one lesion above the threshold value) per PERCIST at the second scan. Melanoma and breast cancer patients were selected from the same patient cohort as in [36], while colorectal cancer patients were scanned at Skåne University Hospital, Sweden during 2022–2023. All scans were clinically indicated. Median age in the test group was 62 years (IQR 23), with 34 (59%) females. The median interval between the PET-CT scans was 14 weeks (IQR 14). The first PET-CT was performed for initial staging (33%), re-staging/recurrence (33%), systematic therapy evaluation (31%), or post-therapy pause (3%). The second PET-CT was primarily performed for systemic therapy evaluation (98%), split between a combination of different systemic treatments (48%) and immunotherapy (50%). Two percent performed the second scan for evaluation of disease after a therapy pause.

Before imaging, patients fasted > 4 h, had blood glucose < 10 mmol/l, and received 4 MBq/kg (maximum 400 MBq) [^18^F]FDG. After 60 min, PET-CT imaging was performed using Discovery MI (GE Healthcare). Scans were acquired from the base of the skull to the mid-thigh, (whole-body for melanoma). The PET acquisition time was 1.5 min per bed position; images were reconstructed using block-sequential regularized expectation maximization (β = 500, 256 × 256 matrix).

A nuclear medicine specialist evaluated baseline and follow-up studies, selecting up to three measurable lesions (the hottest lesions fulfilling PERCIST criteria) per follow-up scan. The manual workflow included:

- **Baseline background activity:** AI-suggested liver VOIs were reviewed and manually adjusted if needed. Aorta VOIs were reviewed in all cases, even when liver VOIs were usable. Correctly positioned background VOIs were registered.
- **Baseline target lesion:** AI-identified target lesions were evaluated. If it was a false positive (uptake considered to be non-malignant), continuation through the AI-generated lesion list was conducted until a measurable target lesion was found. Manual review ensured no normal tissue regions with higher SULpeak than the chosen lesion represented undetected malignancies. The number of target lesions and correctly AI-identified target lesions were recorded.
- **Follow-up background activity:** The same VOI review process was applied as for baseline studies. The AI assessed scan eligibility based on background activity criteria according to PERCIST, which was registered.
- **Follow-up target lesion:** AI-identified target lesions were reviewed. Thresholds were manually adjusted if needed to include lesions with SULpeak below threshold. The total number of lesions, anatomical alignment of lesion pairs, SULpeak changes for lesion pairs, and false-positive AI-suggested lesions were registered.

#### Response assessment

The AI calculated percentage change in SULpeak of the target lesion(s) and suggested PERCIST-based response classification. The system supported manual identification of new or progressive lesions through image alignment and automated quantification of changes in SULpeak.

## Results

### Background activity

AI-suggested liver and aorta VOIs were correctly positioned in all cases, with liver VOI used for threshold calculation. No manual adjustments were needed. Fifty-four patients (93%) met PERCIST quality criteria of similar SUL in the background VOI for both scans. The remaining four, though not meeting strict criteria, were retained to increase sample size for evaluation.

### Target lesions

Target lesions at baseline were identified in 48/58 patients (83%). In 43 cases, the first AI-suggested lesion was correct; in 3, the second suggestion was accurate. Among the 10 patients without measurable lesions, AI correctly identified none in 5 cases. The other 5 had false-positive lesions (hotspots not regarded as malignant by the reviewer), mostly limited to a single lesion.

Across the 58 follow-up studies, 130 lesions were evaluated: 47 lymph node metastases, 37 bone metastases, 19 liver metastases, 19 lung metastases, 3 adrenal gland metastases, 3 breast tumours and 2 melanomas.

**Fig. 4:**
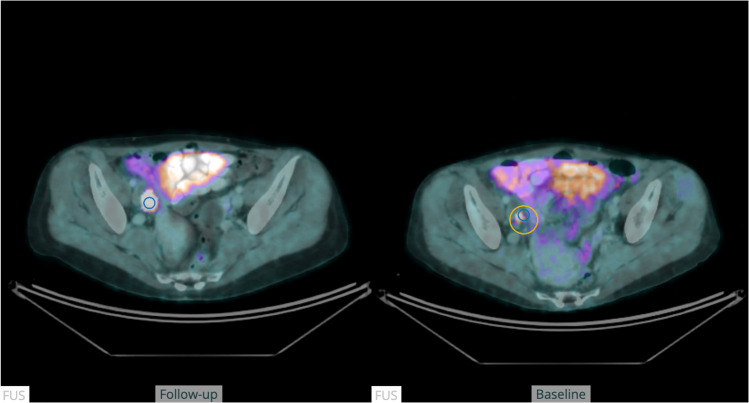
The figure shows a fused transversal slice of a PET-CT scan at baseline (right) and at follow-up (left). The blue circle in the follow-up scan represents the location of the SULpeak of the lesion. The yellow circle in the baseline scan represents the”search volume” for the lesion pairing

**Fig. 5:**
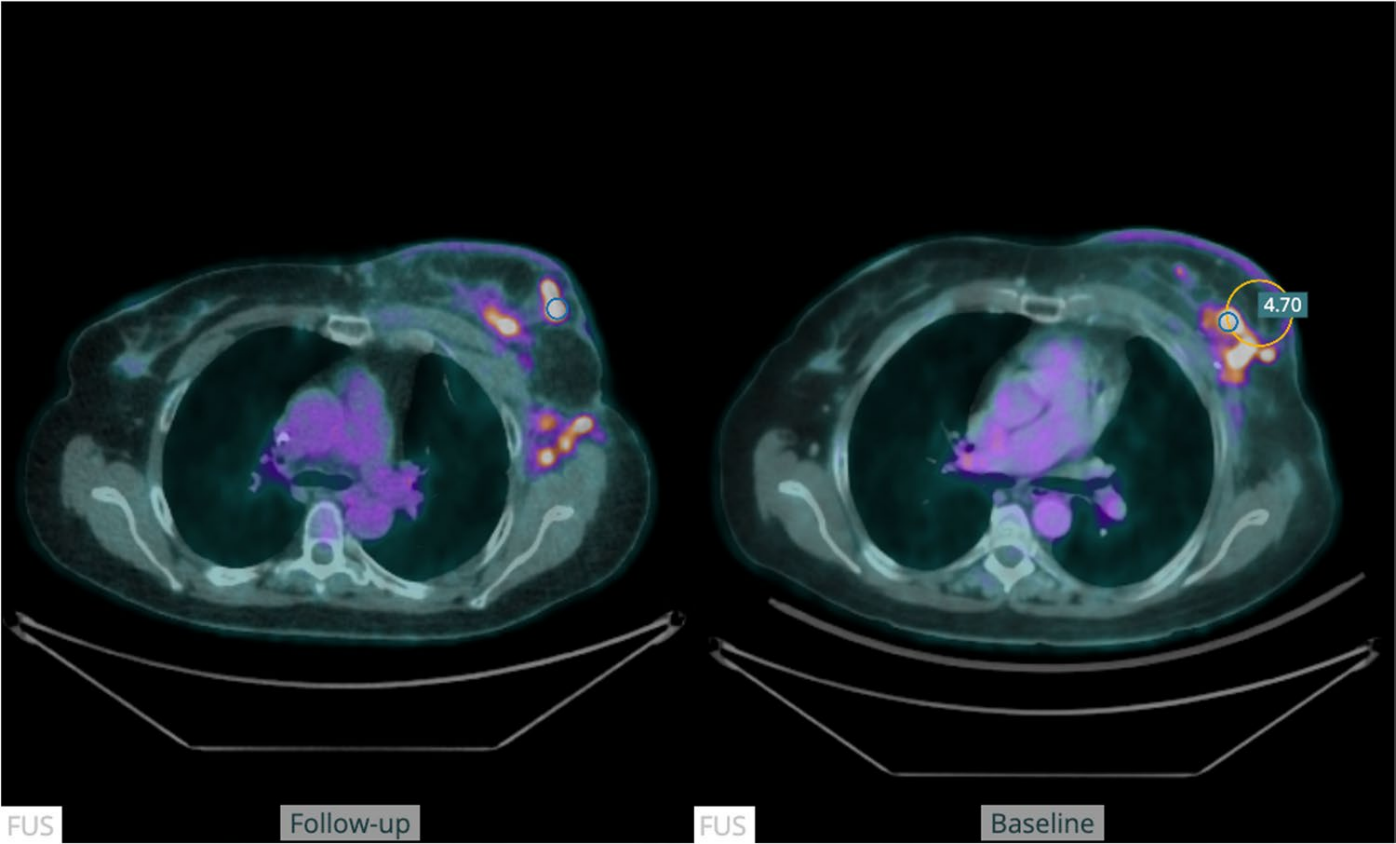
The figure shows a fused transversal slice of a PET-CT scan at baseline (right) and at follow-up (left) in a patient with mixed response in different locations. The blue circle in the follow-up scan represents the location of the SULpeak of the lesion. The yellow circle in the baseline scan represents the”search volume” for the lesion pairing. In this case, the lesion pairing was not accurate

**Fig. 6:**
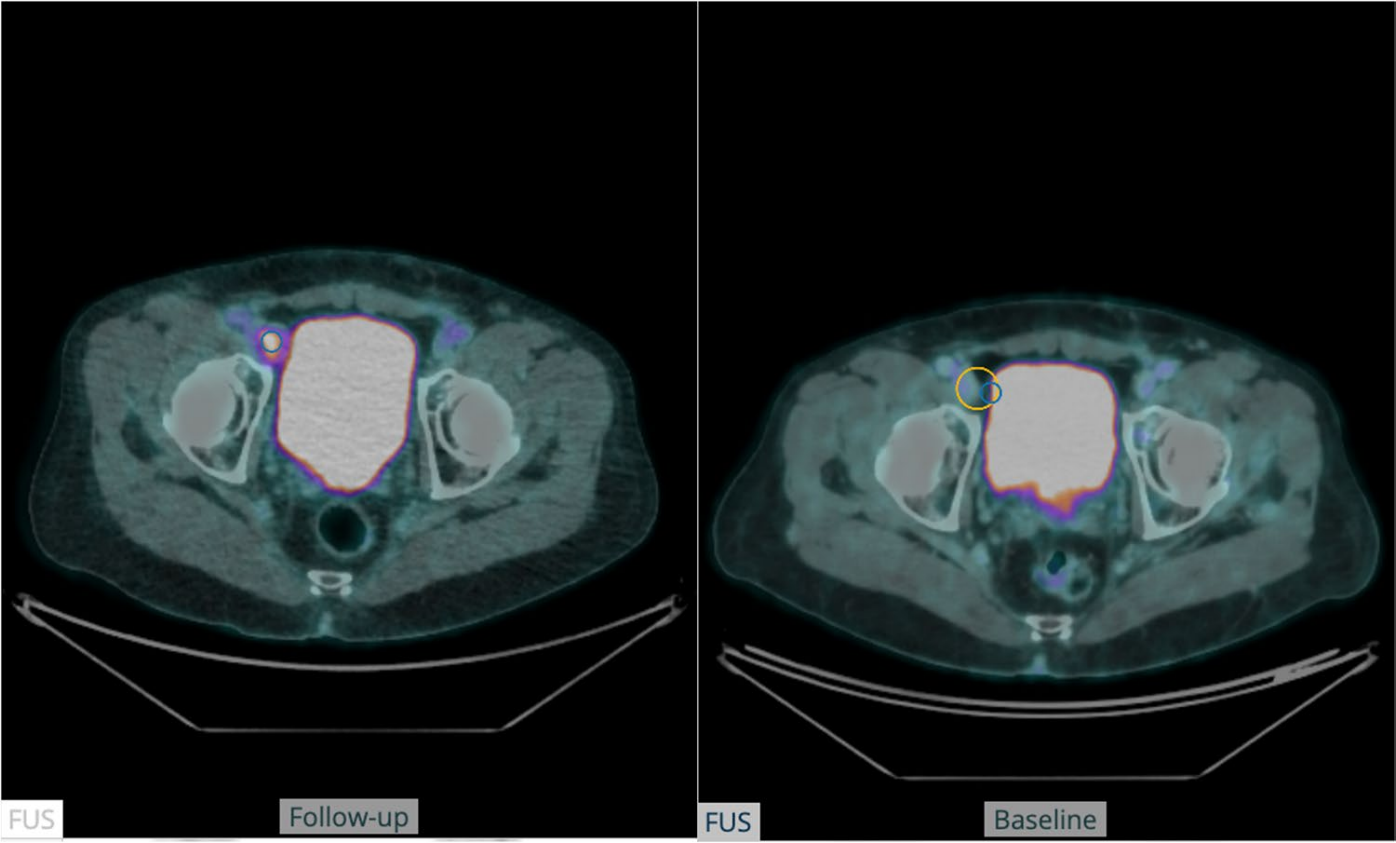
The figure shows a fused transversal slice of a PET-CT scan at baseline (right) and at follow-up (left) in a patient with a lymph node metastasis close to a misclassified part of the urinary bladder, resulting in an incorrect response assessment

The anatomical alignment method demonstrated accurate lesion matching in all cases (Fig. 4). Quantitative assessment of changes in PET tracer activity was accurate in 123/130 lesions (95%). In five cases, although the search region was accurately located, it contained a different lesion as well as the target lesion, leading to an incorrect lesion pairing (Fig. 5). Additionally, in one follow-up study, two lymph node metastases were close to a misclassified part of the urinary bladder, leading to an incorrect assessment (Fig. 6).

During lesion selection, 22 false-positive lesions were encountered and excluded. These occurred in 16 patients (28%). Most were easily identified as non-malignant (e.g. urine contamination, injection sites, and uptake in catheters).

The results of the alignment method for the 181 [^18^F]PSMA-1007 patients are detailed in the Supplementary material.

### Response assessment

Of the 58 patients, 38 were classified as PMD, 16 as SMD, and 4 as PMR. No patients met criteria for CMR due to the inclusion requirement of measurable disease at follow-up. In 35 cases, the response assessment involved manual identification of new lesions or lesions with unequivocal progression, supported by the AI-assisted image alignment and quantitative analysis.

### Review time

For most studies, PERCIST analysis (baseline and follow-up) took less than one minute, as background VOI, target lesion VOIs, calculations of assessability, and changes in SULpeak only required approval. In complex cases with multiple lesions, review time was longer.

## Discussion

This study presents coPERCIST, a semi-automated, AI-assisted implementation of the PERCIST criteria for treatment response assessment in serial PET-CT studies. The complexity of manual PERCIST analysis, requiring background activity quantification, lesion detection, SULpeak thresholding, and longitudinal lesion comparison, has limited widespread adoption. coPERCIST automates these steps while allowing user supervision, ensuring clinical oversight and flexibility in ambiguous cases.

This work was performed within the framework of the EU-funded PREMIO COLLAB project (personalized response monitoring in oncology: co-creating clinical trials in advanced breast cancer), coordinated by Odense University Hospital in Denmark. PREMIO COLLAB aims to improve overall survival and quality of life for patients with metastatic breast cancer by enhancing precision in response monitoring and treatment management. Although the response criteria in PREMIO COLLAB deviate slightly from PERCIST 1.0, coPERCIST is adaptable to both standard and modified protocols, making it suitable for diverse clinical and research applications. This study presents an initial validation across three cancer types. Future validation will be conducted using PET-CT scans from multiple centres within PREMIO COLLAB, focusing exclusively on metastatic breast cancer. Therefore, the current broader validation is essential.

In both clinical studies and routine practice, imaging plays a critical role in evaluating therapeutic efficacy and guiding clinical decisions. Visual assessment remains the most common method, but standardized criteria are crucial, especially in drug development. For CT imaging, the Response Evaluation Criteria in Solid Tumors (RECIST) is widely used, involving measurements of the longest diameter of up to five target lesions. For FDG PET-CT, response criteria include PERCIST, European Organization for Research and Treatment of Cancer (EORTC) and Lugano. Lugano is specific to lymphoma, which is why lymphoma patients were not included in this study. The EORTC criteria relies on SUV rather than SUL. Although the methods show high agreement, PERCIST offers clearer definitions and better reproducibility [37, 38]. In prostate cancer, the response evaluation criteria in PSMA PET-CT (RECIP) have been proposed for PSMA PET-CT, combining total tumour volume and new lesion status to monitor therapeutic efficacy [39].

To date, we are aware of only two semi-automated approaches for PET-based response criteria, one for Lugano and one for PERCIST. The Auto-PERCIST software semi-automates threshold calculation and lesion detection using a threshold based on SULpeak, but manual inspection and lesion selection are still required [40]. No image alignment, lesion tracking or distinguishment of physiological/non-physiological uptakes are incorporated in the Auto-PERCIST software and it does not use AI. Auto-PERCIST was evaluated in a cohort of 30 oncologic patients (diagnoses unspecified), comparing SULpeak changes of the hottest tumour across multiple readers [41]. The intra-class correlation coefficient was 0.87 across all reads and 1.00 when readers selected the same target lesion. Skander et al. [24] presented a semi-automated Lugano response assessment in non-Hodgkin lymphoma patients. Their approach used AI to independently segment suspected lymphoma lesions for both scans, align the image pairs and refine the registered follow-up tumour mask and finally, a rule-based assessment determines the Lugano response. The algorithm output was editable by physicians, and in 81% of the cases, no modifications were needed. The review time was on average 2 min per case. In addition to these studies, Leung et al. [42] developed a promising deep learning method for predicting histopathological complete response from pre- and posttherapy PET-CT imaging. However, no established criteria such as PERCIST were used.

The alignment method in our study, a critical component of longitudinal analysis, demonstrated superior performance compared to previous approaches. Compared to Jönsson et al. [13], our method achieved lower vector magnitude error (Supplementary material), particularly in challenging anatomical regions such as the arms, and is computationally more efficient. Our method required about 2 min per image pair, whereas Jönsson et al.’s method typically took 10–20 min. Detailed runtime statistics are provided in the Supplementary material. Furthermore, unlike prior studies that relied on manual lesion segmentation, our pipeline is fully automated, enhancing scalability and reproducibility. Jönsson et al. evaluated the lesion tracking algorithm using manually segmented tumours, within only 15 patients with metastatic breast cancer and obtained a precision of 93% and a sensitivity of 87% [43] when evaluating the algorithm’s performance by comparing proposed lesions pairs with manually identified ones. The sensitivity decreased for lesions under 1 mL.

To promote transparency and collaboration, coPERCIST is freely available to researchers via the RECOMIA platform (www.recomia.org). The not-for profit organization RECOMIA has made several different AI tools freely available for research purposes. At present, more than 75 research groups from 35 different countries have taken the opportunity to apply our tools to their scans.

We acknowledge several limitations. This evaluation was conducted using PET-CT scans from a single hospital, with a limited patient cohort and three cancer types. Even though the AI models were trained on scans from different institutions, further validation with larger, multi-centre datasets and additional cancer types is necessary. Review time for manual PERCIST analysis was not recorded, so direct comparison with coPERCIST’s review time, typically under one minute, was not possible. Nonetheless, manual PERCIST evaluation is generally more time-consuming.

## Conclusion

This first validation study demonstrates the feasibility of AI-assisted automation of the PERCIST workflow for longitudinal [^18^F]FDG PET-CT response assessment. By integrating advanced image alignment, lesion tracking, and quantitative analysis into a single platform, coPERCIST reduces the manual workload while maintaining promising high accuracy and reproducibility. Its adaptability to both standard and modified PERCIST protocols, combined with open availability to the research community, positions it as a valuable tool for future multicentre trials. Continued validation across broader patient populations and clinical settings will be essential to fully establish its utility in precision oncology.

## Supplementary Information

Below is the link to the electronic supplementary material.

Supplementary file1 (PDF 582 KB)

## Data Availability

The datasets analysed during the current study are available from the corresponding author on reasonable request. coPERCIST is freely available for researchers on the RECOMIA website (www.recomia.org).
