# Supplementary material for: coPERCIST: AI-assisted PET-CT response assessment
Source: Eur J Nucl Med Mol Imaging. 2025 Oct 22;53(4):2314–24. doi: 10.1007/s00259-025-07614-3 (PMC12920726; doi:10.1007/s00259-025-07614-3)
Supplement: Supplementary file 1 — Supplementary file1 (PDF 582 KB) [file 259_2025_7614_MOESM1_ESM.pdf]

# Supplementary material

# Implementation details

## Vertebra segmentation

Individual vertebrae were segmented using a two-stage pipeline. First, a CNN detected vertebral center points; then a 3D U-Net generated instance-level vertebra segmentations centered at these locations.

### Vertebra center detection

The detection model was implemented as a 3D CNN with 11 convolutional layers, each using a kernel size of (3, 3, 3). Each convolutional layer was followed by 3D batch normalization and a leaky ReLU activation. To progressively reduce spatial resolution, the stride was set to 2 for every third convolutional layer, while all other layers used stride 1. Valid padding was applied throughout.

For an input patch of size (82, 82, 82), the model produced two outputs: the estimated probability that the central (8, 8, 8) region contains a vertebral center, and the displacement of the vertebral center relative to the patch center. Since the network was fully convolutional, larger input patches could be processed to obtain predictions at multiple spatial locations simultaneously.

Input CT images were resampled to a voxel size of 1.0 × 1.0 × 1.0 $mm^3$. The intensity values were clamped to the range [-1024, 3096] HU and subsequently normalized to [0, 1].

The model was trained for 600 epochs, with each epoch defined as 100,000 samples. For the probability output, binary cross-entropy loss was applied. For the displacement outputs, mean squared error loss was used, with the modification that displacements within 1 mm of the

ground truth were assigned zero loss. The displacement loss was only applied to positive samples (patches containing a vertebral center).

Training was performed using the NAdam optimizer with an initial learning rate of $8 \times 10^{-5}$, decayed exponentially with a factor of 0.97.

## Vertebra segmentation model

Individual vertebrae were segmented using a 3D UNet–based architecture. The model operated across five resolution levels, combining convolutional blocks, max-pooling for downsampling, and transposed convolutions for upsampling. Feature maps from the encoder path were concatenated with decoder features through skip connections, allowing fine-grained spatial detail to be preserved.

The input consisted of a patch from the CT of size 128 × 128 × 96 voxels, resampled to an effective voxel spacing of 1.0 × 1.0 × 3.0 $mm^3$. Intensities were clipped to the range [–1024, 3096] HU and linearly normalized to [0, 1]. For each voxel, the model predicted one of four classes: target vertebra, superior vertebra, inferior vertebra or background.

Training was performed for 200 epochs, each defined as 20,000 sampled patches. Training samples were generated either as patches centered on a vertebra or as background patches. For vertebra-centered patches, the ground truth labels were relabeled to follow the output convention (target / superior / inferior). The loss function was categorical cross-entropy, optimized with NAdam at an initial learning rate of $8 \times 10^{-5}$, decayed exponentially by a factor of 0.97.

During inference, the segmentation network was applied to patches centered on vertebral locations detected by the preceding detection model. Only the target vertebra class was

retained from the output, and the segmented vertebrae were numbered sequentially from superior to inferior.

## Normal uptake segmentation model

Normal activity was trained on 873 of the studies from a publicly available dataset [1]. Normal uptake was segmented using the same 3D U-Net architecture as described for vertebra segmentation. The network was trained with paired CT and PET input patches, each of size 160 × 160 × 160 voxels, resampled to a voxel spacing of 1.33 × 1.33 × 3.0 $mm^3$.

CT intensities were clipped to [–1024, 3096] HU and linearly normalized to the range [0, 1]. For PET, standardized uptake values (SUV) were calculated, clipped to [0, 100], and normalized to the range [0, 1]. For each voxel, the model predicted one of the following classes: background, brown fat, soft tissue tumor, testes uptake, lung high uptake, vocal cords, brain activity, intestinal tract, urinary tract, heart activity, extraocular muscles, bone high uptake and tonsils salivary glands. Note that some classes representing pathological uptake were included, since corresponding annotations were available from other projects. Including these classes improved the performance of the normal uptake classes as well.

The network was optimized using categorical cross-entropy loss. Optimization was performed with NAdam, starting from a learning rate of $5 \times 10^{-5}$, which decayed exponentially by a factor of 0.97 per epoch.

## Background activity computation

To choose a VOI in the liver the mean SUL (SULmean) and standard deviation of the SUL (SULstd) was computed for each sphere of radius 1.5 cm within the liver volume. The median SULmean

and the median SULstd over these sets were computed. A spherical VOI, located at least 0.75 cm from the liver edge, was selected by minimizing

$| SULmean_{VOI} – median SULmean| + | SULstd_{VOI} – median SULstd |$

This procedure was intended to reduce the influence of outliers, such as focal lesions in the liver, and was found to provide a robust and reproducible placement in practice.

A VOI in the aorta was established in an analogous way.

## Evaluation of the alignment method

The alignment method was evaluated on a dataset consisting of 181 patients who underwent PET-CT imaging at 1 and 2 hours after injection of [$^{18}$F]PSMA-1007. For each patient, there was two PET-CT image pairs: one acquired at 1 hour and the other at 2 hours post-injection.

To assess the quality of the displacement fields, we examined their consistency by computing both the forward transformation $T_{ij}$ from source to target and the inverse transformation $T_{ji}$ from target to source. In an ideal scenario, applying both transformations sequentially should return each voxel to its original position. We defined the Vector Magnitude Error (VME), as introduced by Jönsson et al [2] to quantify the deviation from the original position:

$$VME(x) = \left|x - T_{ji} \circ T_{ij}(x)\right|$$

where x denotes a voxel coordinate in the source image. Lower VME values indicated better consistency and, by extension, higher transformation fidelity.

The VME was evaluated separately for each anatomical region. For each patient, the VME was computed at the voxel level by applying the forward and inverse transformations in both directions (i.e., using each PET-CT image once as the source and once as the target). The resulting voxel-wise VME values were then averaged within each body region to yield a regional VME score. A box plot summarizing the distribution of these scores across all patients is shown in Figure 1S.

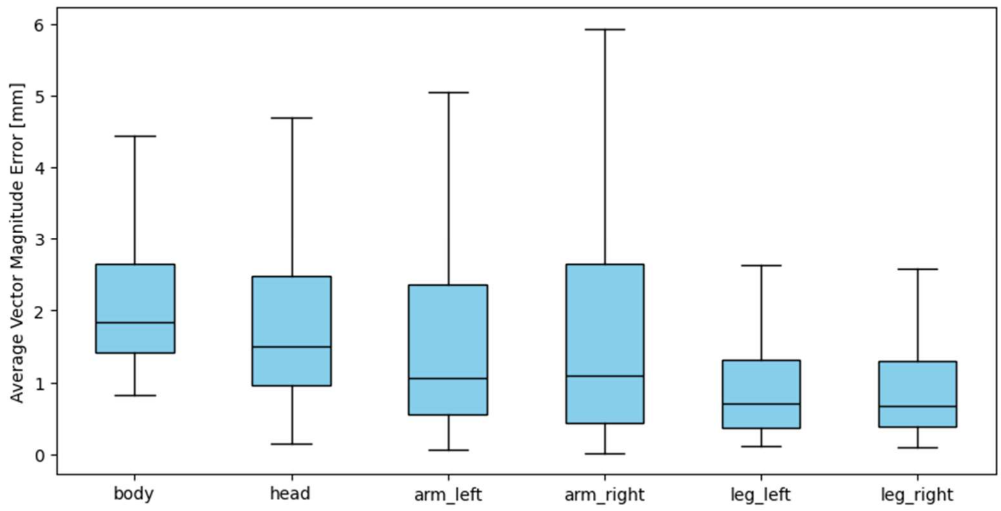


**Figure 1S:** Vector Magnitude Error (VME) by anatomical region. Voxel-level VME values were averaged within each anatomical region to yield a per-region score. Box plots show the distribution of regional VME values across all patients.

## Runtime

The runtime of the alignment procedure and other core automated steps of the PERCIST pipeline was assessed on all 181 patients. All experiments were performed on a workstation equipped with an NVIDIA GeForce RTX 3090 GPU and an AMD Ryzen 9 5900X 12-core CPU.

For the individual segmentation models, the average runtimes (mean ± SD) were as follows:

- Organ Finder: 22 ± 4 seconds per image
- Vertebra segmentation: 96 ± 15 seconds
- Normal activity segmentation: 295 ± 10 seconds

The longer runtime of the normal activity segmentation model is explained by its use of both CT and PET as inputs, combined with a higher input resolution compared to the other segmentation models. As a reference to the segmentations used for the alignment (Organ Finder and vertebra segmentation), the runtime of Totalsegmentator on the same data was 199 ± 10 seconds.

For the full alignment procedure, the mean runtime was 171 ± 15 seconds. Of this, the segmentation stage (including all required models) accounted for 150 ± 12 seconds, while the subsequent alignment step required 18 ± 7 seconds. Since the organ segmentations from Organ Finder are also used for background quantification, the effective runtime for the additional steps required for alignment (beyond organ segmentation) was 120 ± 12 seconds.

## References


1. Gatidis S, Hepp T, Fruh M, La Fougere C, Nikolaou K, Pfannenberg C, et al. A whole-body FDG-PET/CT Dataset with manually annotated Tumor Lesions. Sci Data. 2022;9(1):601
2. Jonsson H, Ekstrom S, Strand R, Pedersen MA, Molin D, Ahlstrom H, et al. An image registration method for voxel-wise analysis of whole-body oncological PET-CT. Sci Rep. 2022;12(1):18768.
